# Attention-Assisted Feature Comparison and Feature Enhancement for Class-Agnostic Counting

**DOI:** 10.3390/s23229126

**Published:** 2023-11-11

**Authors:** Liang Dong, Yian Yu, Di Zhang, Yan Huo

**Affiliations:** College of Information Engineering, Shenyang University, Shenyang 110044, China; yyafengqi123@gmail.com (Y.Y.); zhdizz@163.com (D.Z.);

**Keywords:** class-agnostic counting, crowd counting, few-shot counting, CNN, transformer

## Abstract

In this study, we address the class-agnostic counting (CAC) challenge, aiming to count instances in a query image, using just a few exemplars. Recent research has shifted towards few-shot counting (FSC), which involves counting previously unseen object classes. We present ACECount, an FSC framework that combines attention mechanisms and convolutional neural networks (CNNs). ACECount identifies query image–exemplar similarities, using cross-attention mechanisms, enhances feature representations with a feature attention module, and employs a multi-scale regression head, to handle scale variations in CAC. ACECount’s experiments on theFSC-147 dataset exhibited the expected performance. ACECount achieved a reduction of 0.3 in the mean absolute error (MAE) on the validation set and a reduction of 0.26 on the test set of FSC-147, compared to previous methods. Notably, ACECount also demonstrated convincing performance in class-specific counting (CSC) tasks. Evaluation on crowd and vehicle counting datasets revealed that ACECount surpasses FSC algorithms like GMN, FamNet, SAFECount, LOCA, and SPDCN, in terms of performance. These results highlight the robust dataset generalization capabilities of our proposed algorithm.

## 1. Introduction

Image-based object counting is a prominent research challenge, with diverse applications in fields such as video surveillance, traffic monitoring, and beyond. Counting objects swiftly, especially in scenarios with dense or unevenly distributed objects, poses a challenge, due to limitations in the human visual system. Consequently, the development of a dependable object counting system has emerged as a recent focal point in computer vision research.

**Figure 1:**
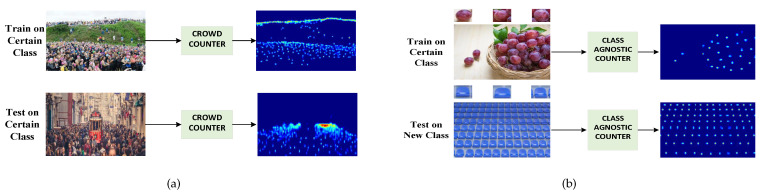
Comparison between class-specific counting and class-agnostic counting: (**a**) only the number of instances of a certain class is counted in both training and inference; (**b**) class-agnostic counter learns the counting method, trains on a certain class, and can count the number of instances of a new class that have never been seen.

Following a comprehensive review of prior research, it is evident that the majority of studies assume the presence of a single class in the training dataset, such as populations, cells, animals, or cars. Examples are illustrated in Figure 1a. This approach is commonly referred to as class-specific counting (CSC). Consequently, when deploying the model, it can only handle task objectives that belong to the same class as the training data. Mainstream approaches typically perform object counting using a series of convolutional neural networks (CNNs) with fixed-size convolution kernels. However, they still require a substantial amount of training data corresponding to specific semantic classes for effective learning.

The few-shot counting (FSC) algorithm, initially proposed by Lu et al. [1], addresses the class-agnostic counting (CAC) problem, as exemplified in Figure 1b. Unlike class-specific counting models, FSC is trained on data encompassing various semantic categories. During the inference process, FSC can effectively capture the visual features of a query image containing new categories by leveraging examples from the corresponding classes, enabling it to count the instances of these categories in a new scene. As articulated by Min in BMNet [2], CAC represents a promising direction in object counting—a shift from merely learning to count objects to learning to count various methods or categories.

Specifically, FSC is designed to determine the quantity of salient objects in an image, regardless of their semantic class, by utilizing a user-provided set of “exemplars” that refer to the specific objects to be counted. Typically, existing FSC methods consist of two main components: feature extraction and matching. Initially, they extract visual features from the exemplars and then compare these features to those extracted from the query image. The outcome of this similarity matching serves as an intermediate representation for inferring object counts.

**Figure 2:**
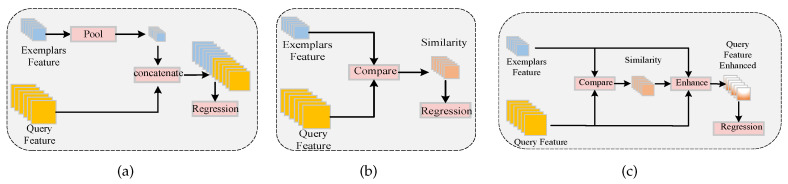
Comparison of existing FSC methods: (**a**) Feature-based approach. In this method, query features are directly combined with pooled exemplar features and used for density map regression; (**b**) Similarity-based approach. This method involves comparing the original features from the query image and exemplars, to generate a feature similarity map, which is then used for regression density map estimation; (**c**) Enhanced similarity-based approach. This approach enhances the original features by incorporating compared similarity features and support features. The resulting enhanced features are then used for regression density map computation.

In essence, FSC comprises two crucial elements: expressive features and a similarity metric. A recent study, BMNet [2], focused on enhancing the similarity metric by applying predefined rules, to identify self-similarity within images. Another approach, SAFECount [3], categorized CAC algorithms into two groups: feature-based and similarity-based approaches. It proposed a fusion of the strengths of both methods, to achieve superior target counting results. As depicted in Figure 2c, this paper extended upon the principles of SAFECount [3] for the CAC task, improving its similarity comparison techniques and feature enhancements. This resulted in a CAC network with enhanced counting performance and more robust generalization capabilities.

This paper presents three key contributions:

Introduction of ACECount: We introduce ACECount, a versatile visual object counting architecture that combines Transformer [4,5] and CNN models, featuring multiple attention mechanisms. ACECount utilizes local and global attention, to explicitly capture features within and between image patches. It incorporates a cross-attention mechanism for similarity to user-provided few-shot examples. Additionally, ACECount employs densely connected multi-scale regression heads, to capture feature similarity at different scales. This comprehensive approach enhances its counting performance.

Cross-Dataset Generality: We thoroughly investigated ACECount’s ability to generalize across different datasets. In the zero-shot scenario, we utilized two learnable tensors to replace exemplar features as inputs. This adaptation enabled the network to disregard irrelevant input portions and transform into a single-class counting network. ACECount demonstrated remarkable performance on both the population count dataset and the FSC-147 dataset under zero-shot conditions, highlighting the effectiveness of our multi-scale regression head for diverse counting tasks.

Ablation Study on CAC Dataset: This study highlights the significant performance improvement achieved by our core module, the feature interaction enhancement module. We also emphasize our careful network structure design and hyperparameter selection, which collectively contribute to ACECount’s outstanding performance in FSC scenarios, establishing it as one of the top-performing solutions.

The content of the following chapters is outlined as follows: in Section 2, we discuss related work, covering the research background and commonly used algorithms in both class-specific object counting and class-agnostic counting tasks; in Section 3, we introduce the ACECount framework and elaborate on the principles and individual roles of each module within the network; in Section 4, we outline the specific experimental program, encompassing an introduction to the dataset, technical algorithm details, comparisons of various methods, and ablation experiments; in Section 5, we provide visualization results for the FSC-147 dataset; in Section 6, we offer a comprehensive summary of the text, highlighting the algorithm’s strengths and weaknesses, along with potential future solutions and directions.

## 2. Related Works

### 2.1. Class-Specific Object Counting

The counting of specific object classes, including crowds [6], vehicles [7], and animals [8], is a common task. Among them, the field of crowd counting has been extensively researched. Traditional methods usually employ machine learning [9,10] for crowd counting. However, this method encounters challenges related to target occlusion and the 2D perspective of images. Consequently, traditional methods are primarily suitable for crowd counting in sparsely populated environments.

With the advent of deep learning, deep-learning-based methods have gradually excelled in crowd counting. The existing crowd counting methods have been predominantly developed around deep learning research.

Deep-learning-based crowd counting methods can be categorized into detection-based counting, regression-based counting, and density-map-based counting. Methods such as [11,12,13] fall into the category of detection-based methods. These methods achieve crowd counting by first detecting individuals and subsequently counting them. Typically, these methods employ object detection algorithms to identify whole bodies, faces, or upper bodies of individuals, with the final count obtained by summing the number of detected instances. However, complex urban scenes or large crowds densely packed together often result in significant occlusion issues. Consequently, such methods struggle to handle occlusion problems within crowds.

To address the challenge of crowd occlusion, Lian and Li et al. proposed the RGBD-Net [14], a detection model that leverages additional depth information. However, this approach necessitates the availability of depth annotation information during training, limiting its applicability.

The regression-based approach [15,16] involves learning the mapping between features extracted from local image patches and their corresponding counts, eliminating the need for object detectors. This approach effectively handles individual occlusion and feature tracking, making it suitable for crowd counting in complex scenes. Unlike detection-based methods, regression-based methods estimate crowd density using an overall crowd description, which is not constrained by high-density scenes. As a result, regression-based methods can more effectively estimate crowd density in complex scenes without requiring explicit object boundaries or individual tracking.

Methods like [17,18,19] are density-map-based, capable of achieving high-performance crowd counting in crowded and complex scenes. Most recent studies in crowd counting have been conducted based on this method. For instance, Pham et al. [18] proposed a density-based method to regress the density map from input images. Additionally, researchers in [17,19] designed multi-column convolutional networks and applied CNNs with various convolutional kernel sizes to extract features at different scales separately, addressing the scale variance problem in crowd counting.

Nonetheless, in any case, these methods face challenges when dealing with object categories not present in the training dataset or counting multiple categories of objects.

### 2.2. Class-Agnostic Counting

Lu et al. were the first to propose class-agnostic counting (CAC) [20,21], and they introduced a generalized matching network called GAM [1]. In this approach, a shared convolutional neural network (CNN) is employed to extract features from both the query and exemplar. These features are then used to calculate similarity, which is subsequently regressed, to generate the density map.

Recognizing that direct regression from connected features might lead to overfitting, recent advancements have focused on explicit similarity modeling. For instance, CFOCNet [21] employs the feature map of the exemplar as a 2D convolutional kernel, convolving the query image with this kernel, to calculate similarity.

To address the counting instance scale challenge encountered by CAC, FamNet [20] introduced a model based on self-similarity matching and introduced the first large CAC dataset, FSC-147. Meanwhile, SAFECount [3] utilizes support features, to enhance the query feature through a feature enhancement module. This enhancement refines the extracted features and leads to regressing a density map with improved accuracy.

Additionally, BMNet [2] incorporated a similarity loss, to supervise the results of similarity matching, drawing inspiration from metric learning. LOCA [22] introduced a novel module for object prototype extraction, combining shape and appearance data with image features iteratively. CounTR [23] streamlined training image generation with a two-stage process, enforcing model use of a specified sample. SPDCN [24] presented scale priori deformable convolution, enhancing counting network efficiency by incorporating sample information for better semantic feature extraction. These existing algorithms demonstrate proficiency in the CAC task but still fall short of achieving the accuracy levels attained by single-class counting algorithms in the class-specific counting (CSC) task.

## 3. Proposed Method

Overview. Few-shot counting (FSC) aims to determine the number of objects in a query image that belong to specific classes. Unlike class-specific counting (CSC), FSC’s primary goal is to learn the capability to count new classes based on existing classes, rather than solely counting objects within predefined classes. In CSC, training data only consist of pixel-level point annotations for object classes. In FSC, object classes are split into two parts: one for training and the other for testing.

We denote the training class as $C_{t}$ and the inference class as $C_{i}$. $C_{t}$ and $C_{i}$ encompass different semantic classes of counted objects, with $C_{t}\cap C_{i}=⌀$. During network training, for each query image from $C_{t}$, represented as $X_{i}^{t}\in R^{H\times W\times 3}$, we provide a certain number of box coordinates to represent the exemplars in the image. These exemplars are denoted as $y_{i}^{t}={b_{i}^{t}}^{K}$, where $K\in \left\{0,1,2,3,\ldots \right\}$, to calculate the number of corresponding categories in the image. Additionally, a ground-truth density map, denoted as $D_{i}^{t}\in R^{H\times W\times 1}$, is provided for supervised network training.

For query images from $C_{i}$, represented as $X_{j}^{i}\in R^{H\times W\times 3}$, only a certain number of exemplars—i.e., $y_{j}^{i}={b_{j}^{i}}^{K}$, where $K\in \left\{0,1,2,3,\ldots \right\}$—are provided, to enable the network to estimate the number of corresponding categories. FSC’s objective is to use a minimal number of exemplars to calculate the number of objects in $C_{i}$. When the user specifies the number of exemplars in a query image as *K*, the task is referred to as K-shot FSC.

Framework Architecture. The ACECount framework comprises six components: the query encoder, the exemplar encoder, the pyramid feature aggregation module (PFA), the similarity comparison module (SCM), the feature attention enhancement module (FAEM), and a multi-scale dense counting regression head (MDCR). The SCM and FAEM collectively form the core part of the framework, known as the feature interaction enhancement module (FIEM).

In broad strokes, the query encoder and exemplar encoder are responsible for extracting features from input queries and exemplars, respectively. The PFA aggregates low-level and high-level features. The SCM integrates image regions with arbitrary given regions through cross-attention, facilitating the comparison of image regions to any given shots and the calculation of their similarity. The FAEM employs spatial and channel attention to enhance the input feature map, augmenting the overall feature representation. The MDCR utilizes densely connected dilated convolution, to expand the receptive fields of the regression head, enhancing multi-scale object recognition and extending network generality for counting different categories.

**Figure 3:**
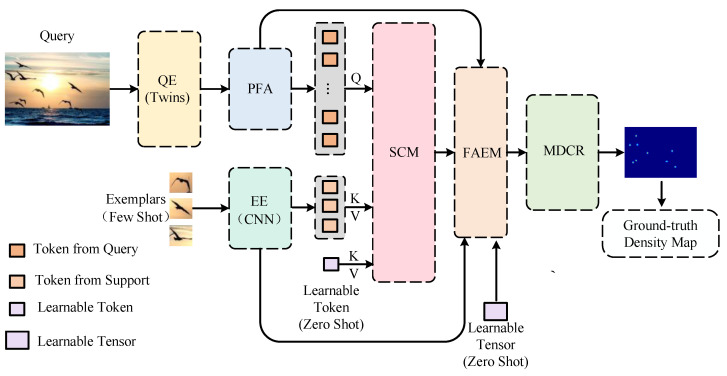
Detailed illustration of our ACECount for object counting.

The network’s versatility for counting different categories is extended, as illustrated in Figure 3.

The pipeline of ACECount involves several key steps. Queries and exemplars are processed through two different encoders, CNN-base and Twins-base, for encoding. Twins reshapes the sequence of vectors back to a 2D feature map, $F_{i}\in R^{C_{i}\times H_{i}\times W_{i}}$, at each downsampling. These features at different stages are sent to the pyramid feature aggregation module (PFA) for feature fusion, to obtain a feature map containing global information, $F_{Q}\in R^{C_{Q}\times H_{Q}\times W_{Q}}$. Meanwhile, the exemplar is transformed into exemplars features, $F_{E}\in R^{M\times D}$, and support features, $F_{S}\in R^{C_{S}\times H_{S}\times W_{S}}$, and it is used as key and value (K and V) by the CNN-base encoder. These are then fed into the PFA along with the query. In the feature attention enhancement module (FAEM), the weight of similar features in *R* is adjusted using the support feature, $F_{S}$, to obtain the reshaped similarity map, $R^{{}^{\prime }}$. This map is then used to enhance the original feature, $F_{Q}$, resulting in the enhanced feature, $F_{R}$. Finally, $F_{R}$ is input into the multi-scale dense counting regression head (MDCR), to obtain the density map and counting results. To simply sum it up:

(1) $$y_{i}=\Phi_{\mathrm{MDCR}}\left(\Phi_{\mathrm{SCM}}\left(\Phi_{\mathrm{QE}}\left(X_{i}\right),\Phi_{PFA}\left(\sum_{l=1}^{n}\Phi_{\mathrm{EE}}\left(S_{i_{l}}^{k}\right)\right)\right),\Phi_{QE}^{n-1}\left(X_{i}\right)\right)\;,\forall k\in \left\{0,1,\ldots ,K\right\},$$

where *k* denotes the number of user-given shots, and *i* denotes the number of query images. In the next section, we will introduce these six modules in detail.

### 3.1. Visual Encoder

Our model’s encoder comprises two components: one following the Twins structure [25], and the other following the CNN structure. These two distinct encoders process two types of input images derived from the same source image: the former is responsible for extracting features from the query images, while the latter focuses on extracting features from the exemplars.

We represent the input image as $I_{Q}\in R^{3\times H\times W}$, where 3, *H*, and *W* denote its channel size, height, and width, respectively. The input exemplar is denoted as $I_{E}\in R^{N\times 3\times K\times K}$, with *N*, 3, and *K* representing its shot number, channel size, height, and width. In the following sections, we will describe these two components separately.

#### 3.1.1. Query Encoder

As transformer technology [5] continues to advance in natural language processing, an increasing number of researchers are exploring its applications in computer vision. Vision in transformers (VIT) [26] was introduced to address the computational complexity of self-attention mechanisms in image processing. Since its introduction, VIT has gradually extended its reach to various computer vision tasks, achieving outstanding results. VIT employs a self-attention layer that divides images into fixed-size patches, embedding them into 1D vector sequences, which serve as input for the self-attention mechanism. These sequences are then linearly transformed, to produce the *K*, *Q*, and *V* matrices, which are used in the self-attention operation. The self-attention calculation process is as follows:

(2) $$\mathrm{Att}\left(Q,K,V\right)=S\left(\frac{QK^{T}}{\sqrt{d}}\right)V,$$

where S(•) denotes softmax, *Q* denotes query, *K* denotes key, *V* denotes value, and *d* denotes the length of the vector.

We utilize Twins-SVT [25], a pyramidal VIT variant, as the query encoder. Twins introduces a novel attention mechanism that combines locally grouped self-attention (LSA) and global sub-sampled attention (GSA) in two combinations, which are then stacked together in multiple modules. This configuration is referred to as spatially separable self-attention (SSSA).

To elaborate, in a given layer *l* of Twins, the feature maps are initially divided into patches, each measuring $k_{1}\times k_{2}$ pixels, and projected into tokens, using a multi-layer perceptron (MLP). At this stage, information exchange only occurs within the sub-window. However, GSA facilitates information flow between different patches, overcoming the limitation of local information processing. Smaller receptive fields could otherwise impact the network’s performance. In the GSA stage, each sub-window produces a single representative through convolutional operations. This representative aggregates critical information from its corresponding sub-window. Subsequently, a self-attention calculation is applied to the representatives of all the sub-windows.

This approach enables sub-windows to communicate with one another through their representatives, capturing global features. Formally, we represent SSSA as follows:

(3) $$\begin{array}{cc} Z_{l}^{\prime } & =\mathrm{LSA}\left(\;\mathrm{LayerNorm}\;\left(Z_{l-1}\right)\right)+Z_{l-1},\\ Z_{l}^{\prime \prime } & =\mathrm{MLP}\left(\;\mathrm{LayerNorm}\;\left(Z_{l}^{\prime }\right)\right)+Z_{l}^{\prime },\\ Z_{l}^{\prime \prime \prime } & =\mathrm{GSA}\left(\;\mathrm{LayerNorm}\;\left(Z_{l}^{\prime \prime }\right)\right)+Z_{l}^{\prime \prime },\\ Z_{l} & =\mathrm{MLP}\left(\;\mathrm{LayerNorm}\;\left(Z_{l}^{\prime \prime \prime }\right)\right)+Z_{l}^{\prime \prime \prime }, \end{array}$$

where LSA(•) represents the self-attentiveness of the local grouping within the sub-window, GSA(•) signifies the global sub-sampling attention obtained by interacting with the representative keys (generated by the sub-sampling function) from each sub-window, and finally, $Z\in R^{k_{1}\times k_{2}}$.

Twins capture fine-grained and short-distance information in the image through the attention operation within the specified sub-window, using LSA. They also capture the global and long-distance information of the image by fusing the attention from various sub-windows through GSA. This mode effectively captures image features while being less computationally and parametrically demanding than the standard self-attention operation, making it easy to deploy.

We incorporate Twins-SVT Large with pre-trained weights from ImageNet, consisting of four stages with 11 layers of GSA and LSA. It is worth noting that the input image undergoes downsampling to $\frac{1}{32}$ of its original size, while the channel dimension expands to 1024. From this architecture, we extract the output feature maps $F_{i}$ from stages 1, 2, and 3, to be used as inputs for the PFA component. The resulting output is represented as follows:

(4) $$F_{i}=\Phi_{QE}\left(X_{i}\right)\in R^{M\times D}.$$

#### 3.1.2. Exemplars Encoder

In the exemplar encoder, when performing K-shot counting (*K* = 0, 1, 2, 3), we employ a standard convolutional structure for feature extraction from the exemplars. Specifically, the exemplar encoder comprises four 3×3 convolutional layers with ReLU activation and average pooling. The object scale remains nearly unchanged as the exemplar images are resized to 3×K×K dimensions. Consequently, we refrain from applying additional operations to the exemplars but instead focus on extracting features and mapping them onto high-dimensional feature maps, using a few shallow CNN layers. The output feature map $F_{S}$ from the penultimate convolutional layer serves as the enhanced support feature input in the FAEM. In scenarios where zero-shot counting is performed (i.e., when no exemplar is provided), to meet the input requirements of the SCM part (*K*, *Q*, and *V*) we incorporate a conditional mechanism to skip this step and replace the feature vector obtained from the feature extractor module with a learnable token, which then serves as the key and value inputs to the SCM:

(5) $$F_{E}=\Phi_{EE}\left(S_{i}^{k}\right)\in R^{M\times D}.$$

### 3.2. Pyramid Feature Aggregation Module

PFA accepts the feature maps $F_{1}$, $F_{2}$, and $F_{3}$ from the final three stages of the Twins network. These maps are down-sampled to $\frac{1}{8}$, $\frac{1}{16}$, and $\frac{1}{32}$ of the original image size, respectively. Additionally, PFA incorporates a top-down feature fusion operation.

As the Twins network becomes deeper, the downsampling factor increases, resulting in high-level features with richer semantic information but at the cost of losing fine-grained details present in low-level features. Simply upsampling with interpolation cannot effectively recover these lost details, including positional, textural, and color information. This limitation makes it challenging to accurately capture the target’s boundary and location solely based on the feature map output of the deep network. This issue is particularly problematic in tasks involving dense object counting.

Numerous approaches have been developed to address this challenge. For instance, the feature pyramid network (FPN) [27] was introduced, to enhance localization accuracy by integrating high-level semantic and low-level features through top-down pathways. PAN [27] further extended FPN, with a bottom-up pathway, to reduce the information path lengths for both low-level and top-level features, facilitating the propagation of accurate signals in low-level features. These architectures have found widespread use in object detection tasks (e.g., the YOLO series [28,29,30,31]), demonstrating the efficacy of multi-scale feature fusion in target detection. Consequently, many object counting approaches have incorporated pyramidal feature fusion modules. However, most of these methods merely perform basic feature concatenation between high-level and low-level features and do not fully exploit the multi-scale information derived from the encoder. This limitation hinders their effectiveness in counting small objects.

**Figure 4:**
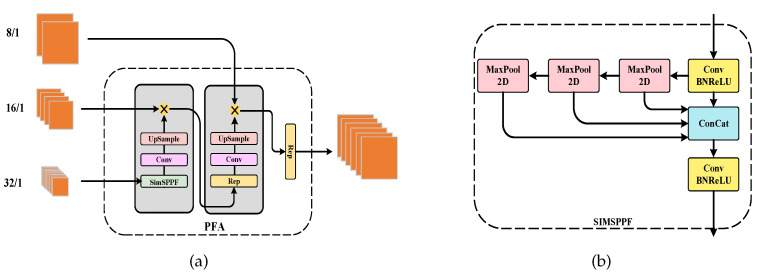
(**a**) overall structure of PFA; (**b**) structure of the SIMSPPF module.

This concept draws inspiration from the remarkable success of YOLOv6 v3.0 [28] in object detection. We have devised a multi-scale aggregation mechanism for integrating information across different scales. Our approach is grounded in the utilization of SIMSPPF [30] and RepBlock [30] as foundational components. An illustration of one of the SIMSPPF structures is provided in Figure 4b, which represents an enhanced iteration of the SPP [29] module. It incorporates the concept of a spatial pyramid, seamlessly merging local and global features, substantially enhancing the feature map’s information representation capacity. This approach effectively mitigates performance degradation, particularly when dealing with substantial size variations in target counting tasks.

Furthermore, RepBlock is employed within the network, featuring a multi-branch topology during training. However, during inference, this multi-branch structure is seamlessly fused into a single 3×3 convolutional layer. The architectural arrangement is illustrated in Figure 4a. Specifically, this module takes as its input the feature maps, $F_{i}$, from the exemplar encoder, each at a different scale. To prevent excessive computational overhead, we reduce the channel dimensions of $F_{i}$, using a 1×1 convolutional layer. $F_{3}$ initially enters the SIMSPPF module, where the feature maps pass through pooling layers of varying kernel sizes before being amalgamated. This approach aims to mitigate image distortion stemming from cropping and scaling operations during data preprocessing, a critical consideration for small object localization and recognition.

The next step involves upsampling the feature maps, using a 3×3 convolutional kernel and bilinear interpolation, to maintain their spatial dimensions consistent with those of $F_{2}$. Subsequently, these up-sampled feature maps are concatenated in the channel dimension. Following the concatenation, the PFA passes through a sequence of layers, including RepBlock, convolutional layers (conv), and upsampling layers, sequentially. Finally, it is combined with the low-level feature, $F_{1}$. This process culminates in the generation of the final output of the PFA component, denoted as $F_{Q}$. To summarize briefly:

(6) $$F_{Q}=Concat\left(F_{i}\left(s\right),F_{i-1}\left(s\right),\ldots ,F_{l+1-\delta }\left(s\right)\right),i\in \left(1,2,3\right),$$

### 3.3. Feature Interaction Enhancement Module

**Figure 5:**
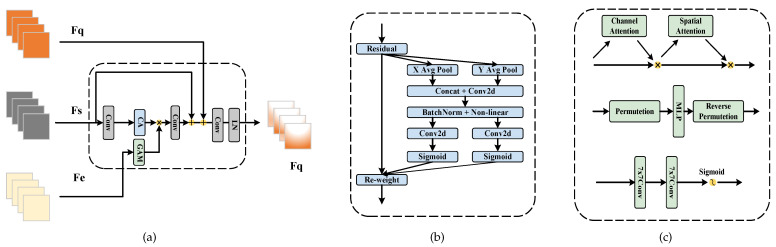
(**a**) overall structure of FAEM; (**b**) structure of the coordinate attention module; (**c**) structure of the global attention mechanism.

As shown in Figure 5a, FSC is typically approached through two mainstream methods: the feature-based approach and the similarity-based approach. In the feature-based approach, query features are compared pointwise, to obtain a feature similarity map, which is then used for regressing a density map via a trained regression head. Examples of such methods include GAM [1], CAFOCNet [21], FAMNet [20], and SAFECount [3].

However, it is important to note that the information contained in similarity maps is often much less comprehensive compared to that in raw features. This limitation makes it challenging to obtain detailed information about the target, hindering the network’s ability to distinguish precise object boundaries. Moreover, the feature-based representation method may lose spatial information from the exemplars, due to the presence of pooling layers, which can adversely impact localization.

In this paper, we propose a similar feature interaction enhancement module that seeks to combine the strengths of both approaches. Specifically, we enhance the original features, denoted as $F_{E}$, from the query image by adjusting the feature weights based on similarity information, represented as $F_{S}$. Subsequently, these enhanced features are used for density map regression. This section’s structure comprises the following two modules.

#### 3.3.1. Similarity Comparison Module

Lu et al. proposed in GAM [1] that the counting problem can be transformed into a matching problem by leveraging the self-similarity property inherent in images, which naturally occurs in object counting tasks. This approach allows for counting arbitrary objects in a class-agnostic manner.

For this paper, we employed a series of standard transformer decoder structures, to assess the self-similarity of images. Specifically, we treated query image features as queries, and we used an MLP to transform exemplar features into two distinct projections: key and value. The cross-attention mechanism in the transformer decoder computed the similarity between query and value, comparing arbitrary regions in the image to a user-defined number of exemplar shots, to measure their similarity. We used standard 2-layer transformer decoder structures. We projected each $\frac{1}{8}$-size feature map pixel from the input image into a sequence. This sequence entered an embedding layer with a hidden dimension of 512. After passing through the SCM, the vector sequences were converted back into 2D feature maps:

(7) $$F_{SCM}=\Phi_{SCM}\left(\frac{\left(F_{PFA}W^{Q}\right){\left(F_{CNN}W^{K}\right)}^{T}}{\sqrt{d}}\right)\left(F_{CNN}W^{V}\right)\in R^{M\times D}.$$

By comparing the query and key in the similarity space, the model was asked to select the appropriate information from the features automatically and, with the help of the features of the few shots given by the user, the score *R* of the user’s region of interest was calculated by comparison.

#### 3.3.2. Feature Attention Enhancement Module

Channel attention involves compressing the feature map into a 1D representation along the spatial dimension and performing attention operations along its channel dimension. This module enables the model to prioritize regions of interest while suppressing attention in non-interesting regions. For instance, CBAM [32] employs a large-scale convolutional kernel to compute an attention map across both channel and spatial dimensions. It then multiplies the feature map with this attention map, to adaptively optimize the features. This approach allows for capturing long-range dependencies along one spatial direction while preserving precise location information along the other. The resulting feature maps are transformed into direction-aware and position-sensitive attention maps, which can be complementarily applied to the input feature maps, to enhance the representation of the object of interest.

More specifically, when we consider the features $F_{Q}$, $F_{E}$, and $F_{S}$ obtained from the query image and exemplars, along with the feature similarity graph *R* generated from SCM, our proposed FAEM aims to enhance the original features. FAEM initially processes the support features, $F_{S}$, through the GAM module. Leveraging spatial and channel attention mechanisms, the network emphasizes the salient objects in the image (i.e., exemplars). The feature similarity map *R* is then dimensionally reduced, using a 1×1 convolutional layer, to reduce computational complexity. Subsequently, the feature map attention region is reshaped, and the coordinate attention module derives the reshaped similarity map, denoted as $R^{{}^{\prime }}$. Following this, $R^{{}^{\prime }}$ is combined with the query feature map, $F_{Q}$, to enhance the relevant feature dimensions of the k-shot image provided by the user. $F_{Q}$, $F_{S}$, and $F_{E}$ are input into FAEM, and the attention distribution is adjusted, based on the weights of $F_{Q}$ in spatial and channel dimensions, utilizing two attention modules, CA and GAM, respectively. This component effectively blends the CNN and the attention mechanism, to make the network more focused on our task objectives.

The realization of this part can be divided into two steps:

Similar attention enhancement: In this step, we aggregate the support features, $F_{S}$, by incorporating coordinate attention (CA) [33] and the global attention mechanism (GAM) [34]. The structures are illustrated in Figure 5b,c. Essentially, in relation to the exemplar features $F_{E}$ provided by the user, regions with higher similarity in *F* should be assigned greater weights in both spatial and channel dimensions. The spatial and channel attention mechanism accomplishes this weighted aggregation.

Original image feature enhancement: In this step, we incorporate the enhanced similarity graph, $R^{{}^{\prime }}$, into the original features of the query image, $F_{Q}$, using a shallow convolutional layer. This process enhances the weights of the feature maps that are close to the target class in exemplars. The enhancement is accomplished through a straightforward matrix addition before entering the convolutional layer.

### 3.4. Multi-Scale Dense Counting Regression Head

**Figure 6:**
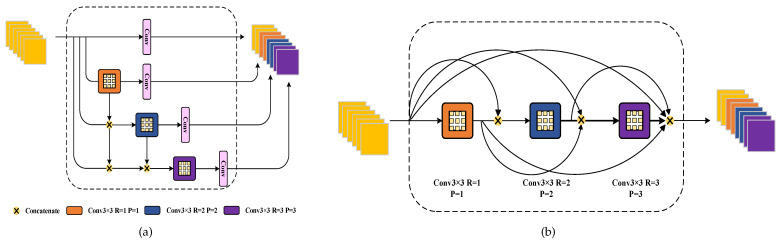
Architecture for MDCR: (**a**) the architecture consists of a series of densely connected cavity convolutions; (**b**) is the equivalent topology of MDCR, which consists of a series of stacked densely sampled Dconv layers.

We have obtained global and multi-scale information through PFA and enhanced the features corresponding to exemplars using FAEM, which employs feature similarity and attention mechanisms. Next, we require a robust regression head, to accurately estimate the density map. The structure of this regression head is depicted in Figure 6a.

In general, a standard CNN followed by a pooling layer can be employed for counting tasks, and this architecture is often stacked multiple times, to generate a density map in the decoder part (e.g., SAFECount). However, this approach continuously reduces the spatial resolution of the feature map, leading to the loss of detailed information and a diminished ability to detect small targets.

**Figure 7:**
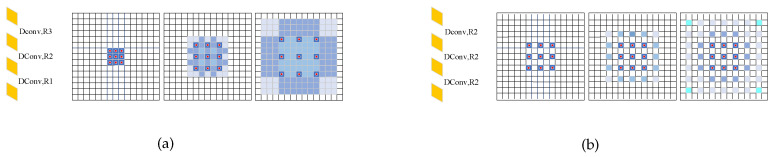
The receptive field on the input feature map visible to each layer of the final convolution kernel varies with different dilation rate selections. In the visual representation, blue squares denote the receptive field, and red dots represent the actual pixels involved in the convolution operation: (**a**) when a fixed dilation rate of 2 is used, it results in the gridding effect; (**b**) using fixed dilation rates of 1, 2, and 3 does not lead to the gridding effect.

To make better use of the information within the feature map, it is essential to expand the perceptual field of the regression head. Directly using larger fixed-size convolutional kernels would significantly increase the computational demands of the network. Dilated convolutional (DConv) layers [35] provide an excellent solution to enhancing the perceptual field without increasing computational complexity. A common practice is to stack DConv layers together [35]. However, this can lead to the gridding effect of DConv layers, where, after passing through multiple convolutional layers, some pixels in the feature map may be missed in subsequent convolutions, as illustrated in Figure 7b. Therefore, the dilation rate of DConv layers must be carefully chosen, to ensure that the convolution captures a larger receptive field while avoiding the gridding effect and the loss of information at a distance.

In ACECount, ensuring the density map’s resolution requires using a final feature map size that is $\frac{1}{8}$ of the input image’s size. When dealing with class-independent counting, where target scale variations can be substantial and a diverse range of targets is encountered, having a large receptive field becomes essential. To address this, we employ a dense ASPP structure with the smallest feasible expansion coefficient. Specifically, the multi-scale dense counting regression head (MDCR) module consists of three parallel Dconv layers denoted as $C_{i}$ (*i* = 1, 2, 3), accompanied by a 1×1 convolutional branch functioning as a shortcut. The output of each of these expanded convolutions is then passed through subsequent convolutional layers before being concatenated and processed through the DConvs with varying expansions (*R* = 1, 2, 3). The overall structure of this module can be simplified, to resemble Figure 6b, equivalent to a sequence of cascading null convolutions. Subsequently, we reduce the feature map’s channel count through two straightforward convolutional layers, resulting in a one-channel density heatmap. The number of targets is obtained by simply summing the heatmap values. The computational load of this spatial processing component is defined as follows:

(8) $$K=K_{3,d=1}+K_{3,d=2}+K_{3,d=3}-3,$$

where 3 denotes convolution kernel size and *d* denotes dilation rates.

We denote this part as follows:

(9) $$y_{i}=\Phi_{MDCR}\left(C_{1},C_{2},C_{3},S\right)\in R^{1\times M\times D}.$$

### 3.5. Zero-Shot Counting

In the context of zero-shot counting, where the training data either lacks exemplar annotations or does not utilize them, we replace the exemplar features, $F_{E}$, and support features, $F_{S}$, with two learnable tensors ($T_{1}$ and $T_{2}$). Specifically, $T_{1}$ is used as both the key and value inputs within the SCM component, while $T_{2}$ serves as the input for the FAEM segment’s support features. This innovative approach enables the network to seamlessly adapt to different values of ‘k’ in k-shot counting scenarios, thereby enhancing its performance in zero-shot counting tasks.

### 3.6. Loss Function

We use a loss function that is common in population counting, which comes from DM-Count [16] and is represented by a weighted sum of count loss, optimal transmission (OT) loss, and total variance (TV) loss. Count loss: let ${∥\cdot ∥}_{1}$ be the $L_{1}$ parameterization of the vector, ${∥z∥}_{1}$,$∥\hat{z}{∥}_{1}$ denote the predicted density map number and the actual value number, respectively, and the count loss requires the values of ${∥z∥}_{1}$, $∥\hat{z}{∥}_{1}$ to be as close as possible. The final count loss is defined by the absolute value of the difference between the two only:

(10) $$l_{c}\left(z,\hat{z}\right)=∥∥z∥{}_{1}-∥\hat{z}∥{}_{1}∥.$$

Optimal transmission loss: *z*, $\hat{z}$ are both non-normalized density functions, which can be converted into probability density functions by treating them as their respective sums. OT loss can measure the similarity between two density maps and, simultaneously, help the counting model obtain a firm fit, providing an effective gradient to train the network to minimize the distribution gap between the predicted density map and the ground truth. It is defined as follows:

(11) $$l_{OT}\left(z,\hat{z}\right)=W\left(\frac{z}{{∥z∥}_{1}},\frac{\hat{z}}{∥\hat{z}{∥}_{1}}\right)=\langle \alpha^{*},\frac{z}{{∥z∥}_{1}}\rangle +\langle \beta^{*},\frac{\hat{z}}{∥\hat{z}{∥}_{1}}\rangle .$$

Total variance: Wang et al. (2020) [16] pointed out that OT loss approximates target dense regions well but produces inaccurate results for regions with low target density. In this regard, TV loss is used to stabilize the supervised signal and increase the stability of the training process. It is defined as follows:

(12) $$l_{TV}\left(z,\hat{z}\right)={∥\frac{z}{{∥z∥}_{1}}-\frac{\hat{z}}{∥\hat{z}{∥}_{1}}∥}_{TV}=\frac{1}{2}{∥\frac{z}{{∥z∥}_{1}}-\frac{\hat{z}}{∥\hat{z}{∥}_{1}}∥}_{1}.$$

Finally, the DM loss function is a weighted sum of the counting loss, OT loss, and TV loss, which is expressed as

(13) $$l_{dm}=l_{1}\left(z,\hat{z}\right)+\lambda_{1}l_{oT}\left(z,\hat{z}\right)+\lambda_{2}l_{TV}\left(z,\hat{z}\right),$$

where *z*, $\hat{z}$ denote the target number of estimated and true density maps, respectively, and $\lambda_{1}$ and $\lambda_{2}$ are adjustable hyperparameters.

## 4. Experiments

### 4.1. Dataset

We conducted experiments using our framework on five benchmark datasets: FSC-147 [20], CARPK [34], ShanghaiTech Part A [17], ShanghaiTech Part B [17], and UCF-QNRF [16]. These experiments included class-agnostic counting on FSC-147, class-specific crowd counting on ShanghaiTech, UCF-QNRF, and class-specific car counting on CARPK. These datasets vary in terms of counting classes, image resolution ratios, the number of objects, object density, and color spaces.

For the FSC-147 dataset during training, we used all the bounding box annotation information as exemplars in the 3-shots case. In the i-shots case (where i = 1, 2), i boxes were randomly selected from the three boxes, as exemplars input.

The ShanghaiTech, and UCF-QNRF datasets do not provide bounding box annotations. In these cases, we trained the datasets in a zero-shot setting, using a learnable tensor instead of exemplars input.

In the case of CARPK, which provides bounding box annotations, we obtained the point annotation information by computing the centroid coordinates of the bounding boxes, to generate the true value density map. Exemplars were obtained by randomly selecting the bounding boxes, and the remaining settings were kept consistent with the training of the FSC-147 dataset.

FSC-147 is one of the few available datasets for few-shot counting (FSC). It features a multi-category setup with 147 classes and 6135 images. Each image includes three bounding boxes representing instances of objects from count categories specified by exemplars. The dataset’s data distribution is as follows: the training set comprises 89 classes and 3659 images, while the validation and test sets consist of 1286 and 1190 images, respectively, including 29 additional disjoint classes. The object count per image ranges from 7 to 3701, with the number of images containing over 1000 objects being relatively low, averaging 56. The FSC-147 dataset presents two primary challenges. First, most prior algorithms were designed for counting a specific target class, whereas FSC-147 encompasses diverse categories and necessitates the ability to handle unseen categories during inference. Second, the dataset spans various scenarios, demanding algorithms to effectively filter out irrelevant context interference in task goals.

The ShanghaiTech dataset comprises two parts, A and B. Part A includes images collected from the internet, exhibiting a wide range of population density variations. The data distribution for Part A consists of 300 images in the training set and 128 images in the test set. By contrast, Part B contains images captured by surveillance cameras on Shanghai streets, featuring significant intrascene scale and density variations. Part B’s data distribution comprises 400 images in the training set and 316 images in the test set.

UCF-QNRF images were sourced from various websites and exhibit diversity in scenes and sizes. These images primarily contain objects at a small scale. The dataset consists of a total of 1535 images, with 1201 in the training set and 334 in the test set. No validation set is specified.

CARPK serves as a class-specific car counting benchmark commonly used to assess the generalizability of FSC network datasets. It includes 1448 images of cars captured by drones in four different parking lots, encompassing a total of 90,000 cars.

### 4.2. Implementation Detail

For data augmentation, we uniformly applied random cropping to each image. To be precise, the FSC-147 and ShanghaiTech images were randomly cropped to 256×256 pixels, while images from UCF-QNRF were cropped to 512×512 pixels. Randomized horizontal flipping operations were also performed on the datasets above.

In the network’s MDCR, the significant increase in feature map channels due to dense connectivity required strategies to manage model size and computational load. To tackle this, we used multiple 1×1 convolutional layers to reduce the number of feature channels in the intermediate stage, thereby effectively reducing the overall model’s parameter count.

During training, we froze the weights of the query encoder part of the network and only updated the parameters of the remaining components. After 50 epochs of training, the weights of the encoder part were unfrozen. This strategy preserved the generic semantic information obtained from pre-training on ImageNet, which was loaded into the encoder part of the network. We employed the AdamW [36] optimizer with a batch size 16, a well-suited choice for transformer-based models. The initial learning rate was set to $1\times 10^{-5}$, and $L_{2}$ regularization of 0.0001 was employed, to prevent overfitting. In the class-specific counting dataset, we initialized the network parameters with weights trained on the FSC-147 dataset and subsequently fine-tuned them on the corresponding CSC dataset. Simultaneously, we kept the weights of the backbone part frozen.

We conducted our experiments within the PyTorch framework, employing an NVIDIA GeForce RTX 3090 environment.

### 4.3. Evaluation Metrics

Reviewing the previous work in object counting, we adopted two commonly used counting methods as our evaluation metrics: mean absolute error (MAE) and root mean squared error (RMSE). They are defined as follows:

(14) $$\mathrm{MAE}=\frac{1}{N_{l}}\sum_{i=1}^{N_{I}}\left|C_{i}-C_{i}^{GT}\right|,$$

(15) $$\mathrm{RMSE}=\sqrt{\frac{1}{N_{I}}\sum_{i=1}^{N_{I}}{\left(C_{i}-C_{i}^{GT}\right)}^{2}},$$

where $C_{i}$ denotes the number of people predicted by ACECount, $C_{i}^{GT}$ denotes the actual number of people in each image, and *N* indicates the total number of images.

### 4.4. Compared to Different Methods

#### 4.4.1. Class-Agnostic Counting

**Figure 8:**
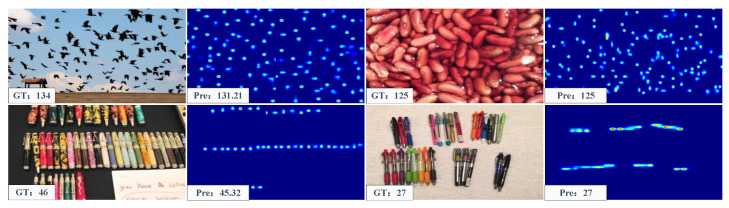
Visualization of FSC-147. The first and third columns are the input images and the second and fourth columns are the corresponding density maps.

Training Configuration on Class-Agnostic Counting. The dimensions of the query images and exemplar images were set to 256×256 and 64×64 for H×W and $H_{E}\times W_{E}$, respectively. As presented in Table 1, ACECount demonstrated great performance in terms of zero-shot and few-shot counting, surpassing previous methods, particularly in the validation set results. The visualisation results are displayed in Figure 8.

Comparing Methods. We evaluated and compared the proposed ACECount model to existing CAC methods on the FSC-147 dataset: Under the few-shot training setting, we compared CFOCNet [16], FSOD [37], Counting-DETR [38], BMNet [2], and FamNet [20]. For BMNet [2], we used the variant BMNet+ [2] that implements both the core components, i.e., the self-similarity module and the dynamic similarity metric. Additionally, for more comparisons, we also tested SAFECount [3], which applies a similar design to our work, i.e., combining feature-based and similarity-based approaches. We also compared RepRPN-C [39] and RCC [40] under the zero-shot training setting, demonstrating that our method can perform well under zero-shot counting as well.

Performance on FSC-147. Table 1 lists the quantitative results for FSC-147. Specifically, ACECount demonstrated a clear advantage over some methods (CFOCNet, FSOD, Counting-DETR, BMNet+, and FamNet) that are either based solely on features or rely on a single similarity metric. Compared to SAFECount, which is also based on similarity enhancement, ACECount was able to more accurately distinguish the boundary contours of the target object and adapt to scale variations in target objects, identifying smaller targets. ACECount achieved an MAE of 14.93 and an RMSE of 50.11 on the validation set, and an MAE of 14.06 and an RMSE of 83.51 on the test set. It is important to note that SAFECount and BMNet+ already served as a strong baseline for ACECount. This performance improvement was mainly attributed to our network’s enhanced FIEM, which effectively leveraged the attention mechanism and combined similarity and raw feature information, to optimize the use of the encoder’s initial features.

**Table 1:** Comparison with the CAC algorithm on the FSC-147 dataset.

Method	Venue	Shots	Val		Test	
			**MAE**	**MSE**	**MAE**	**MSE**
RepRPN-C [39]	ACCV23	0	31.69	100.31	28.32	128.76
RCC [40]	Arxiv22	0	20.39	64.62	21.64	103.47
ACECount (ours)	2023	0	17.92	68.31	16.16	105.23
CFOCNet [16]	WACV21	3	21.19	61.41	22.10	112.71
FSOD [37]	CVPR20	3	36.36	115.00	32.53	140.65
FamNet [20]	CVPR21	3	23.75	69.07	22.08	99.54
BMNet+ [2]	CVPR22	3	15.74	58.53	14.62	91.83
Counting-DETR [38]	ECCV22	3	-	-	16.79	123.56
SAFECount [3]	WAC23	3	15.28	47.20	14.32	83.54
ACECount (ours)	2023	3	14.93	50.11	14.06	83.51

#### 4.4.2. Class-Specific Object Counting

**Figure 9:**
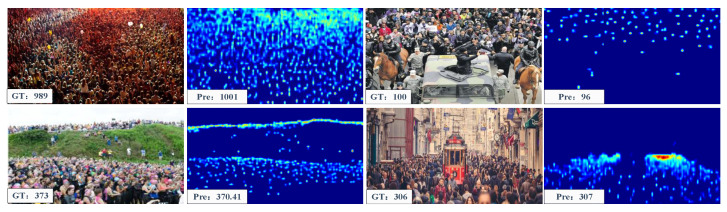
Visualization results of ACECount used for object counting in the ShanghaiTech Part A dataset. The first and third columns are the input images and the second and fourth columns are the corresponding density maps.

Training Configuration on Class-Specific Counting. In our experiments on the crowd counting dataset, we utilized a zero-shot counting training configuration. We compared our method to two categories of competitors: single-class crowd counting methods and FSC methods. Conversely, for the vehicle counting dataset, we adopted a three-shot training configuration. Our results demonstrated that our method outperforms all FSC methods in crowd counting and matches the performance of State-of-the-Art single-class crowd counting methods. Furthermore, our approach excels in vehicle counting. It is worth noting that our model was not originally designed for specific car counting or crowd counting tasks. the visualisation results are displayed in Figure 9.

Performance on ShanghaiTech. The FSC approach lacks the ability to handle multi-scale targets and struggles to detect small-scale crowd targets when transitioning from the FSC-147 dataset to the crowd counting dataset. Thanks to our MDCR module, our network efficiently identifies and locates small pixel targets. ACECount outperforms all FSC methods, in terms of CSC task results, and it is on a par with State-of-the-Art single-class crowd counting methods.

Performance on UCF-QNRF. The results are presented in Table 2. Our method consistently outperformed other CAC methods. This is attributable to the detailed information contained within our PFA, facilitating the detection of small objects. Furthermore, our MDCR excelled in capturing multi-scale features and global contextual information, enabling accurate crowd size regression. In comparison to class-specific crowd counting algorithms, our MAE and RMSE were surpassed only by the previous top-performing algorithms.

Performance on CARPK. The model, initially trained on the FSC-147 dataset, underwent fine-tuning on CARPK and was then compared to dedicated car counting models and other general counting models. The results are presented in Table 3.

### 4.5. Ablation Study

For this section, we performed comprehensive ablation experiments on the proposed network structure, using the FSC-147 dataset. These experiments aimed to demonstrate the effectiveness of our proposed modules and to assess each module’s contribution to the network. Additionally, we conducted extensive ablation studies, to select hyperparameters, determine the optimal size and number of regression head convolutions, investigate the impact of weight freezing during training, and evaluate the effectiveness of the core modules, SCM and FAEM.

#### 4.5.1. Pyramid Feature Aggregation Module

In Table 4, we present the network’s performance when it received features from different encoder stages, aiming to assess the effectiveness of the progressive feature aggregation (PFA) module. In the deeper layers of the encoder, the network tended to lose intricate details, retaining primarily the image’s high-level semantic features, which could be detrimental to our ultimate goal. $F_{1}$, $F_{2}$, and $F_{3}$ were derived from the output features of the three phases within the backbone network. Simultaneously leveraging features from all three phases enabled the aggregation of comprehensive information across various scales, resulting in improved counting performance. The experimental findings highlighted that not only the incorporation of multi-stage features impacts network performance but also that the organization of these features plays a pivotal role. Our designed PFA module consistently outperformed both the straightforward concatenation of the three output features (as indicated in line 4) and the feature fusion using the ASPP approach (line 5).

#### 4.5.2. Multi-Scale Columns

As shown in Table 5, we assessed the impact of varying the number of columns in the DConv layer structure within the MDCR on counting performance. $C_{1}$, $C_{2}$, and $C_{3}$ denote the successive null convolutions, as depicted in Figure 6a. By default, all the experiments included a shortcut path comprising a 1×1 convolution.

Intriguingly, this structural design primarily aimed to enhance generalization across diverse datasets. Notably, on the FSC dataset, employing a single $C_{3}$ column also yielded favorable results for the network. However, a more practical approach involved the comprehensive aggregation of all columns, leading to enhanced network performance. We achieved an MAE and an MSE of 14.9 and 50.1, respectively, on the validation set. These results compare favorably to the best outcomes of 16.7 and 62.1, obtained using only a single column of DConv layers, reflecting improvements of −1.8 and −12, respectively. This underscores the indispensability of MDCR.

Simultaneously, we explored different stacking strategies for DConv layers. While stacking three DConv layers identically and setting the dilation rates to 2 offered some performance improvement, it introduced meshing issues, as discussed in Section 4.5.4, ultimately resulting in performance degradation, compared to the approach outlined in this paper. Stacking DConvs with varying dilation rates in depth mitigated this problem, albeit with negligible improvements in the context of object counting tasks.

#### 4.5.3. Freezing Backbone

As we mentioned in the training details in Section 4.5, we undertook the operation of freezing the encoder weights first and then unfroze them later, during training. This aimed to preserve as much as possible the high-level semantic information of various categories learned in ImageNet contained in the weights, so that the network was more general and could achieve effective counting in the face of different categories. We investigated the effect of freezing the weights on the network’s performance, and the results are shown in Table 6. It can be seen intuitively that the operation of freezing weights did bring some performance improvement to our network. The MAE at the time of testing was reduced from 16.3 to 14.0.

#### 4.5.4. Component Analysis of ACECount

We comprehensively assessed the network’s designed structures on the FSC-147 dataset, to gauge each component’s influence on the network’s overall performance. We derived the count structure measures by integrating the components: namely, PFA, SCM, FEAM, and MDCR. The findings are summarized in Table 7, revealing notable performance enhancements attributed to incorporating PFA, SCM, FEAM, and MDCR structures. PFA, SCM, and FEAM structures were the most influential in augmenting the model’s performance. This demonstrates the core modules’ usefulness in our design for the target counting task.

## 5. Visualization in the FSC-147 Dataset

**Figure 10:**
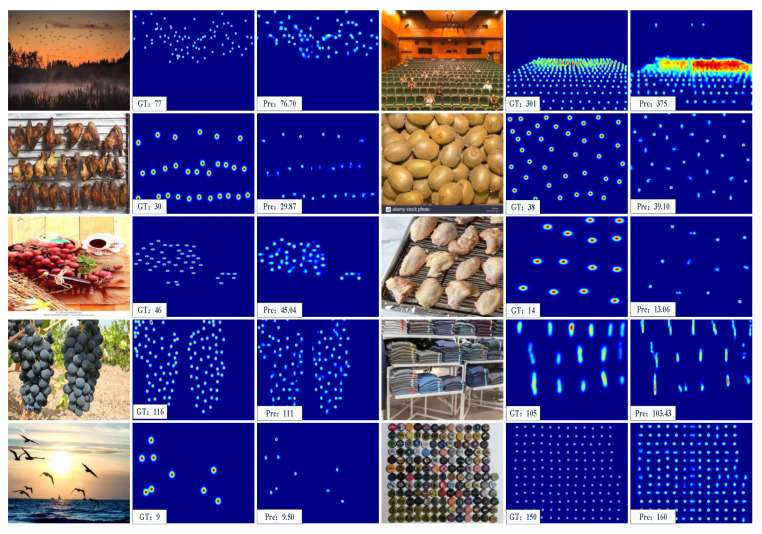
Some qualitative results of ACECount at the three-shot counting setting.

In Figure 10, we provide visualizations of ACECount’s performance in three-shot counting. ACECount stands out compared to methods solely reliant on features or a single similarity metric. It excels in the task of separating densely overlapping objects into individual entities, as demonstrated in Figure 10. This not only leads to more accurate counting results for such objects but also enables the network to precisely distinguish object boundaries, clarify overlapping object contours, and provide valuable information about target localization, including the number of targets, their density distributions, and precise positions. Additionally, ACECount proves effective for counting smaller and densely arranged targets, such as the flying birds and folded clothes in Figure 10, offering accurate counts even for objects that are challenging to discern with the naked eye.

## 6. Conclusions

In this paper, we present a streamlined pipeline based on the FSC approach, customized for the CAC task. Our pipeline comprises six crucial modules: a query encoder designed to effectively capture global contextual information using pyramid vision converters; an exemplar encoder for extracting exemplar features and supporting features through CNN layers; PFA for comprehensive utilization of coarse-to-fine information; SCM for comparing image similarity information through self-attention mechanisms; FEAM for enhancing raw features with spatial and channel attention mechanisms; and the regression head MDCR for achieving a multi-scale receptive field. Our approach demonstrates significant advantages across various popular benchmarks. Nevertheless, irrelevant background information can hinder the counting network’s performance. Background elements resembling the target object may lead to erroneous identifications. To address this concern, we plan to incorporate relevant additional information (such as scale, texture, and color) into the counting network in future research. This enhancement will enable the network to extract semantic features of objects akin to the provided example and to eliminate irrelevant background interference. Additionally, in the future, we aim to enhance the network and extend its application into the realm of weakly supervised learning, which has the potential to significantly reduce the requirement for laborious labeling of image data in goal counting algorithms. In summary, our approach provides a robust baseline for future research on FSC algorithms and has the potential for application in other counting tasks.

## Figures and Tables

**Table 2:** Comparison with the CSC algorithm on the class-specific crowd counting dataset.

	Method	Part A		Part B		UCF-QNRF	
		**MAE**	**MSE**	**MAE**	**MSE**	**MAE**	**MSE**
1	CAN [41]	62.3	100	7.8	12.2	107.0	183.0
	SFCN [42]	59.7	95.7	7.4	11.8	102.0	171.0
	SUA-Fully [43]	66.9	125.6	12.3	17.9	119.2	213.3
	BCCT [44]	53.1	82.2	7.3	11.3	83.8	143.4
	DM-Count [16]	59.7	95.7	7.4	11.8	85.6	148.3
	GL [45]	61.3	95.4	7.3	11.1	84.3	147.5
2	GMN [1]	95.8	-	-	-	-	-
	FamNet [20]	159.1	-	24.90	-	-	-
	SAFECount [3]	73.70	-	9.98	-	-	-
	ACECount (ours)	59.4	96.7	7.2	11.5	90.3	156.2

1. Single-class crowd counting methods; 2. Few-shot counting methods.

**Table 3:** Comparison with the CSC algorithm on the class-specific car counting dataset.

Method	Venue	CARPK	
		**MAE**	**MSE**
LOCA [22]	Arxiv23	9.9	12.5
FamNet [20]	CVPR21	18.1	33.6
PDEM [46]	CVPR19	6.7	8.5
BMNet [2]	CVPR22	9.6	14.8
GAM [1]	CVPR19	7.4	9.9
SPDCN [24]	BMVC22	10.0	14.1
BMNet+ [2]	CVPR22	5.7	7.8
ACECount (ours)	2023	5.6	7.5

**Table 4:** Feature aggregation in PFA.

Strategy	VAL		TEST	
	**MAE**	**MSE**	**MAE**	**MSE**
$F_{1}$	21.3	69.8	21.9	105.3
$F_{2}$	16.7	62.1	17.2	96.9
$F_{3}$	16.8	60.0	15.9	94.3
$F_{1}^{\prime }+F_{2}^{\prime }+F_{3}^{\prime }$	15.5	56.4	14.7	89.2
$F_{1}^{\prime \prime }+F_{2}^{\prime \prime }+F_{3}^{\prime \prime }$	15.3	52.5	14.3	85.5
$F_{1}+F_{2}+F_{3}$	14.9	50.1	14.0	83.5

$F_{1}^{\prime }+F_{2}^{\prime }+F_{3}^{\prime }$ means that the three levels of features are directly stitched together. $F_{1}^{\prime \prime }+F_{2}^{\prime \prime }+F_{3}^{\prime \prime }$ denotes the aggregation of the three levels of features in an ASPP structure. $F_{1}+F_{2}+F_{3}$ denotes the aggregation of the three levels of features in the form of PFA in ACECount.

**Table 5:** Multi-scale columns in MDCR.

Strategy	VAL		TEST	
	**MAE**	**MSE**	**MAE**	**MSE**
$C_{1}$	15.7	58.3	16.1	97.3
$C_{2}$	16.6	59.5	15.3	96.4
$C_{3}$	16.3	60.2	14.9	98.1
$C_{1}^{\prime }+C_{2}^{\prime }+C_{3}^{\prime }$	15.8	56.2	16.0	94.5
$C_{1}^{\prime \prime }+C_{2}^{\prime \prime }+C_{3}^{\prime \prime }$	15.5	50.6	15.2	86.3
$C_{1}+C_{2}+C_{3}$	14.9	50.1	14.0	83.5

$C_{1}^{\prime }+C_{2}^{\prime }+C_{3}^{\prime }$ means stacking three R = 2 DConv layers in depth. $C_{1}^{\prime \prime }+C_{2}^{\prime \prime }+C_{3}^{\prime \prime }$ denotes the stacking in depth of the three DConvs with R = 1, 2, 3, respectively. $C_{1}+C_{2}+C_{3}$ means the structure used in ACECount.

**Table 6:** Freezing Backbone.

Strategy	VAL		TEST	
	**MAE**	**MSE**	**MAE**	**MSE**
Unfreezing	17.8	69.4	16.3	102.9
Freezing	14.9	50.1	14.0	83.5

**Table 7:** Pipeline component analysis of ACECount.

SCM	FEAM	PFA	MDCR	VAL		TEST	
				**MAE**	**MSE**	**MAE**	**MSE**
✓	×	×	×	25.2	70.3	26.1	106.5
✓	✓	×	×	18.6	60.5	18.4	97.6
✓	✓	✓	×	15.8	54.0	16.0	89.6
✓	✓	✓	✓	14.9	50.1	14.0	83.5

A ✓ indicates that the structure is used, and × indicates that the structure is not used.

## Data Availability

We used publicly available datasets.
